# *Wolbachia* is not all about sex: male-feminizing *Wolbachia* alters the leafhopper *Zyginidia pullula* transcriptome in a mainly sex-independent manner

**DOI:** 10.3389/fmicb.2014.00430

**Published:** 2014-09-01

**Authors:** Hosseinali Asgharian, Peter L. Chang, Peter J. Mazzoglio, Ilaria Negri

**Affiliations:** ^1^Program in Molecular and Computational Biology, Department of Biological Sciences, University of Southern CaliforniaLos Angeles, CA, USA; ^2^DISAFA - Department of Agricultural, Forest and Food Sciences, University of TorinoGrugliasco (TO), Italy

**Keywords:** *Wolbachia* infection, male feminization, principal component analysis (PCA), *Zyginidia pullula* transcriptome, transcriptome de novo assembly, host-symbiont interactions

## Abstract

*Wolbachia* causes the feminization of chromosomally male embryos in several species of crustaceans and insects, including the leafhopper *Zyginidia pullula*. In contrast to the relatively well-established ecological aspects of male feminization (e.g., sex ratio distortion and its consequences), the underlying molecular mechanisms remain understudied and unclear. We embarked on an exploratory study to investigate the extent and nature of *Wolbachia*'s effect on gene expression pattern in *Z. pullula*. We sequenced whole transcriptomes from *Wolbachia*-infected and uninfected adults. 18147 loci were assembled *de novo*, including homologs of several *Drosophila* sex determination genes. A number of transcripts were flagged as candidate *Wolbachia* sequences. Despite the resemblance of *Wolbachia*-infected chromosomal males to uninfected and infected chromosomal females in terms of sexual morphology and behavior, principal component analysis revealed that gene expression patterns did not follow these sexual phenotype categories. The principal components generated by differentially expressed genes specified a strong sex-independent *Wolbachia* effect, followed by a weaker *Wolbachia-sexual* karyotype interaction effect. Approaches to further examine the molecular mechanism of *Wolbachia*-host interactions have been suggested based on the presented findings.

## Introduction

*Wolbachia* is an intracellular symbiont alpha-proteobacterium that infects a wide range of arthropods and nematodes (Schulenburg et al., 2000; Werren et al., 2008). It is often transmitted vertically from females through the eggs to their future progeny; although, horizontal transfer between hosts has also been documented (Werren et al., 1995; Cordaux et al., 2001). Studying the mechanism of *Wolbachia*-host interactions is fascinating for many reasons. *Wolbachia* is capable of inducing several intriguing sex-related phenotypes in its hosts, including male killing (MK), in which infected males die during embryonic or larval stages; male feminization (MF), that is the development of genetic males into females; thelytokous parthenogenesis (TP) in which infected virgin females produce daughters. All of these phenotypes distort the progeny sex ratio in favor of females thus ensuring higher transmission rate of *Wolbachia* to the next generation of hosts (Werren et al., 2008; White et al., 2013). Another fascinating effect of the infection is cytoplasmic incompatibility between gametes (CI), which results in aberrant or considerably reduced offspring production, if uninfected females mate with infected males, or if the parents are infected with different *Wolbachia* strains (Werren et al., 2008; White et al., 2013). In this case, infected females possess a reproductive advantage compared to uninfected ones, and this again ensures the spreading of *Wolbachia* into the host population. Fast transition between the four phenotypes in the course of the coevolution of *Wolbachia* and its hosts hints that similar molecular mechanisms might underlie the apparently different effects (Ma et al., 2014). Due to its enormous host range, *Wolbachia* may have played a crucial role in the evolution of sex determination system and reproductive strategies in arthropods (Cordaux et al., 2011; Awrahman et al., 2014; Ma et al., 2014).

Various approaches have been employed to investigate the *Wolbachia*-host interactions in naturally infected and uninfected strains (Hoffmann et al., 1990; Negri et al., 2006; Riparbelli et al., 2012), experimentally inoculated cell lines (Noda et al., 2002; Xi et al., 2008), and antibiotic treated specimens (Hoffmann et al., 1990; Casiraghi et al., 2002). Although *Wolbachia* is an obligate intracellular symbiont natuarally, protocols have been developed to keep it viable in cell-free media for days; however, no replication occurs in the extracellular phase (Rasgon et al., 2006; Gamston and Rasgon, 2007). The experimental/analytical techniques comprised a wide range including classical crossing and fecundity measurements (e.g., Hoffmann et al., 1990; Dunn et al., 2006), microscopic approaches (*in situ* hybridizations, electron microscope and immunohistochemical techniques for bacterium detection inside hosts and cells, tissues, etc.) (e.g., Negri et al., 2008; Fischer et al., 2011), gene expression analysis (e.g., Xi et al., 2008; Kremer et al., 2009, 2012; Hughes et al., 2011; Chevalier et al., 2012; Darby et al., 2012; Liu et al., 2014), bioinformatic genome sequence annotation and functional prediction (e.g., Wu et al., 2004; Foster et al., 2005; Klasson et al., 2008), and mathematical modeling of the ecological consequences of CI or sex ratio distortion (e.g., Taylor, 1990; Turelli, 1994). Despite all these efforts, a coherent mechanistic story of *Wolbachia*'s effect is still lacking. The picture is incomplete even for CI which occurs in *Drosophila* and is the most extensively studied *Wolbachia*-induced phenotype; although, cytoskeleton reorganization and asynchrony in nuclear envelope break down and chromosomal condensation of male and female pronuclei after fertilization have been implicated in the process (Serbus et al., 2008; Werren et al., 2008). The other three phenomena are less well understood. TP seems to result from induction of diploidy in species with a haplodiploid sex determination system by production and development of diploid eggs; that is achieved by altering meiosis to produce diploid gametes (Weeks and Breeuwer, 2001), the abortion of the first mitotic division after chromosomal duplication (Pannebakker et al., 2004), or the fusion of the two haploid nuclei after first mitosis of induced eggs (Gottlieb et al., 2002). The molecular bases of MK and MF are least understood but they are suspected to share certain components as MK is often the result of a lethal and incomplete attempt at feminization of genetic male embryos (Werren et al., 2008). The most direct mechanistic evidence comes from the study of male killing *Wolbachia* in the moth *Ostrinia scapulalis* showing that it overrides the karyotypic signal in genetic males to produce the female *dsx* isoform (Sugimoto and Ishikawa, 2012). This suggests that *Wolbachia* impacts the sex determination pathway at or above *dsx*. Apart from this direct effect on the pivotal sex determining gene *dsx*, MK or MF *Wolbachia* infection is reported to be accompanied with defective chromatin remodeling (Riparbelli et al., 2012), induction of host immune response (Chevalier et al., 2012), and epigenetic reprogramming of the host (Negri et al., 2009a).

*Zyginidia pullula* is a leafhopper with XX/XO male heterogametic sex determination system in which *Wolbachia* causes feminization of chromosomal males (Negri et al., 2006). Infected female leafhoppers are morphologically indistinguishable from uninfected females; but feminized chromosomal males have an intersex phenotype i.e., they have the upper pygofer appendages, a typical male secondary sexual feature. These appendages show varying degrees of development, from being fully developed in some specimens to being a barely recognizable stump in others (Negri et al., 2006). Feminized males with upper pygofer appendages reduced to a stump have ovaries morphologically similar to uninfected females, whereas those with prominent appendages possess malformed and probably less functional ovaries (Negri et al., 2008). The “degree of feminization” has been shown to be correlated with *Wolbachia* density in the host tissues in several systems (Jaenike, 2009). We have previously reported that *Wolbachia* instigates epigenetic reprogramming of *Z. pullula* (Negri et al., 2009a,b) and probably interacts with the insect hormone biosynthesis pathway to stimulate the production of feminizing hormones (Negri et al., 2010; Negri, 2012). In this study, whole transcriptomes of male and female *Zygindia* samples (*Wolbachia*-infected and uninfected) were analyzed with Illumina deep sequencing technique, in order to understand the scope and nature of the *Wolbachia*-induced change in the host gene expression profile. Our initial idea was that if male feminization is the main consequence of *Wolbachia* infection, transcriptomes from the three female types (uninfected females, infected females and feminized males) should resemble each other and be different from the only phenotypically male group (uninfected males). In fact, we decided to test the hypothesis that sex reversal is *Wolbachia*'s main effect at the transcriptome level. Were this confirmed, we would proceed to identify differentially expressed genes between the two sexual phenotype groups.

## Methods

### *Zyginidia* specimens

34 overwintering females of *Z. pullula* were collected in the same grass field in north Italy; and were reared individually in the laboratory as described in Negri et al. (2006). Overwintering females have often mated with several males (rarely with only one). By carefully examining the progeny, *Wolbachia-*infected (i.e., all female brood) and uninfected (i.e., male and female brood) lines were identified. *Wolbachia* infection was then confirmed by PCR on the mothers and randomly chosen samples from the brood as described in Negri et al. (2006). Morphological investigation as to the presence or absence of upper pygofer appendages lead us to separate feminized males from genetic females in the all-female (i.e., *Wolbachia-*infected) lines, and males and females in the uninfected lines. Males from uninfected lines were mated to the physiologically female progeny of the infected lines (consisting of genetic females and males) at each generation to produce the next generation of infected females (and feminized males). This backcrossing to uninfected males was done for at least three generations in the lab. Fifty adults from each of the four different categories of uninfected females (F), uninfected males (M), infected females (FW) and feminized (infected) males (MW) were pooled together for RNA sequencing.

### cDNA library preparation and short-read sequencing

cDNA libraries were made from male and female specimens of infected and uninfected leafhopper lines. Infected males are phenotypically intersex and exhibit different degrees of feminization depending on the concentration of *Wolbachia*, ranging from individuals with functional ovaries to individuals with female secondary sexual characters, but possessing testes. We used thoroughly feminized infected males for RNA extraction. RNA purification, cDNA synthesis and Illumina library construction were performed using the protocols of Mortazavi et al. (2008), with the following modifications: total RNA, mRNA and DNA were quantified using a Qubit fluorometer (Invitrogen); mRNA fragmentation was performed using Fragmentation Reagent (Ambion) for a 3 min and 50 s incubation at 70°C and subsequently cleaned through an RNA cleanup kit (Zymo Research); additional DNA and gel purification steps were conducted using Clean and Concentrator kits (Zymo Research). Each sample library was sequenced as pair-ended 76-base reads on an Illumina Genome Analyzer II.

### *De novo* transcriptome assembly and expression level calculation

Due to the sensitive nature of *de novo* assembly, it is critical that the reads used to generate contigs have the highest sequencing quality. Reads were removed from consideration in the *de novo* assembly if they had a terminal *phred* (Ewing and Green, 1998) quality value less than 15, or contained more than 2 unknown nucleotides (i.e., *N*). Reads were also filtered due to similarities to known PCR primer and Illumina Adapter sequences. Using the reads pooled from all of the four samples that were not filtered out, the *de novo* assembly program Velvet (version 1.0.15) (Zerbino and Birney, 2008) was used in conjunction with a custom post-processing algorithm capable of retaining information from alternative splices (Sze et al., 2012) to assemble short reads into contigs, using sequence overlap information until the contigs could no longer be extended. Velvet was run under the following settings with a kmer length of 35: -cov_cutoff auto -max_branch_length 0 -max_divergence 0 -max_gap_count 0 -read_trkg yes. Sequenced reads that were kept as pairs and not filtered out together or separately were treated as “-shortPaired” with insert length of 175 bases and standard deviation of 75 bases. Single end reads that were not filtered out were treated as “-short.”

With the set of *de novo* assembled sequences serving as a reference, reads from each of the individual samples were mapped using the Burrows-Wheeler Aligner (BWA) (Li and Durbin, 2009). The number of reads that mapped to the contigs of each gene was tabulated and normalized to calculate FPKM (Fragments Per Kilobase Of Exon Per Million Fragments Mapped). Additional normalization among all samples was performed using the TMM protocol (Trimmed Mean of M-values) outlined in Robinson and Oshlack (2010), which takes into account differences in overall RNA populations across samples and is one of several methods used to evaluate RNA sequencing data. Normalization was implemented using the edgeR package in R (Robinson et al., 2010). All statistical analyses and graphs evaluating consistency between samples were produced using R v2.13.0 (R Development Core Team, 2011).

### Gene functional annotation and classification

Blast2GO v.2 (Götz et al., 2008) and WEGO (Ye et al., 2006) were used to obtain Gene Ontology (GO) annotations. Genes were also annotated using a BLASTX search (Altschul et al., 1990) (Expected value <1.00e-05) to the nr protein database available from GenBank as well as to the set of protein sequences available from the *Drosophila melanogaster* 5.34 and the pea aphid *Acyrthosiphon pisum* 2.1 releases. We chose the annotation with the highest BLAST score as long as the span of the alignment was greater than 80% of the length of the contig under query. For genes that did not report any hits, we lowered the minimum span to 40% of the length, choosing the annotation with the highest BLAST score having Expected value <1.00e-05.

### Principal component analysis of gene expression values

Expression values were cleaned of extreme outliers, quartile-normalized and log-transformed before they were used for PCA. To make sure the result were not artifacts of the data preparation method, PCA was repeated on the raw (not normalized, not log-transformed) expression values as well as after several different outlier-filtering and normalization strategies. These statistical procedures were done in SAS 9.3.

## Results

### Short-read sequencing and *de novo* assembly

The mRNA population was analyzed with Illumina deep sequencing of male and female *Zygindia* samples with and without *Wolbachia* infection. The pooled data from all samples had a total of 50 M pair-ended reads that were 76 bases long. All Illumina sequences are available for download at the NCBI Short Read Archive under the BioProject PRJNA171390. After sequences were filtered based on quality and matches to adapter and primer sequences, the 38 M reads from all four samples were pooled together and run through Velvet and the post-processing algorithm. Eventually, 18,147 loci and a total of 27,236 transcripts were assembled; multiple transcripts of a locus pertained often to different splicing isoforms and occasionally to largely differentiated alleles. The transcripts ranged in lengths from 291 bp to 15,389 bp, with mean and median lengths of 1006 bp and 702 bp, respectively. This assembly included a fairly large number of long transcripts: 25% were longer than 1250 bp and 10% were longer than 2000 bp. Of the 18,147 loci, 14,068 (77.5%) had a single isoform and the remaining 22.5% had multiple ones. Transcripts within a locus were subsequently collapsed into a single “representative locus sequence” by using ClustalW to run a multiple sequence alignment and identifying the locus consensus sequence. Mean and median lengths of consensus sequences were 900 bp and 618 bp, respectively. The total length of all loci consensus sequences was 16.3 Mb.

### Gene functional annotation and classification

6946 loci, corresponding to 38% of the entire dataset, were Gene Ontology annotated with Blast2GO. The consensus sequences were also aligned using a BLASTX search to the nr protein database available from GenBank as well as to the set of protein sequences available from Flybase and the aphid genome. Table 1 shows the proportion of cases that resulted in a hit where the length of the alignment was greater than 80% or 40% of the length of the query (leafhopper sequence). One might very crudely attribute the 80% alignment span hits to true genic homology and the 40% alignment span hits to conserved domains.

**Table 1:** **Summary statistics of *Zyginidia* transcripts homology search**.

	**40% homology length**	**80% homology length**
Genbank	60%	39%
Flybase	48%	26%
Pea aphid	81%	32%

40% homology length indicates that the length of the homologous segment covers at least 40% of the query (leafhopper) sequence. A correspondingly similar definition applies to the 80% homology length category. The percent values in the table cells show what percent out of all loci (18147) fit each criterion when Blasted against the designated dataset.

**Figure 1:**
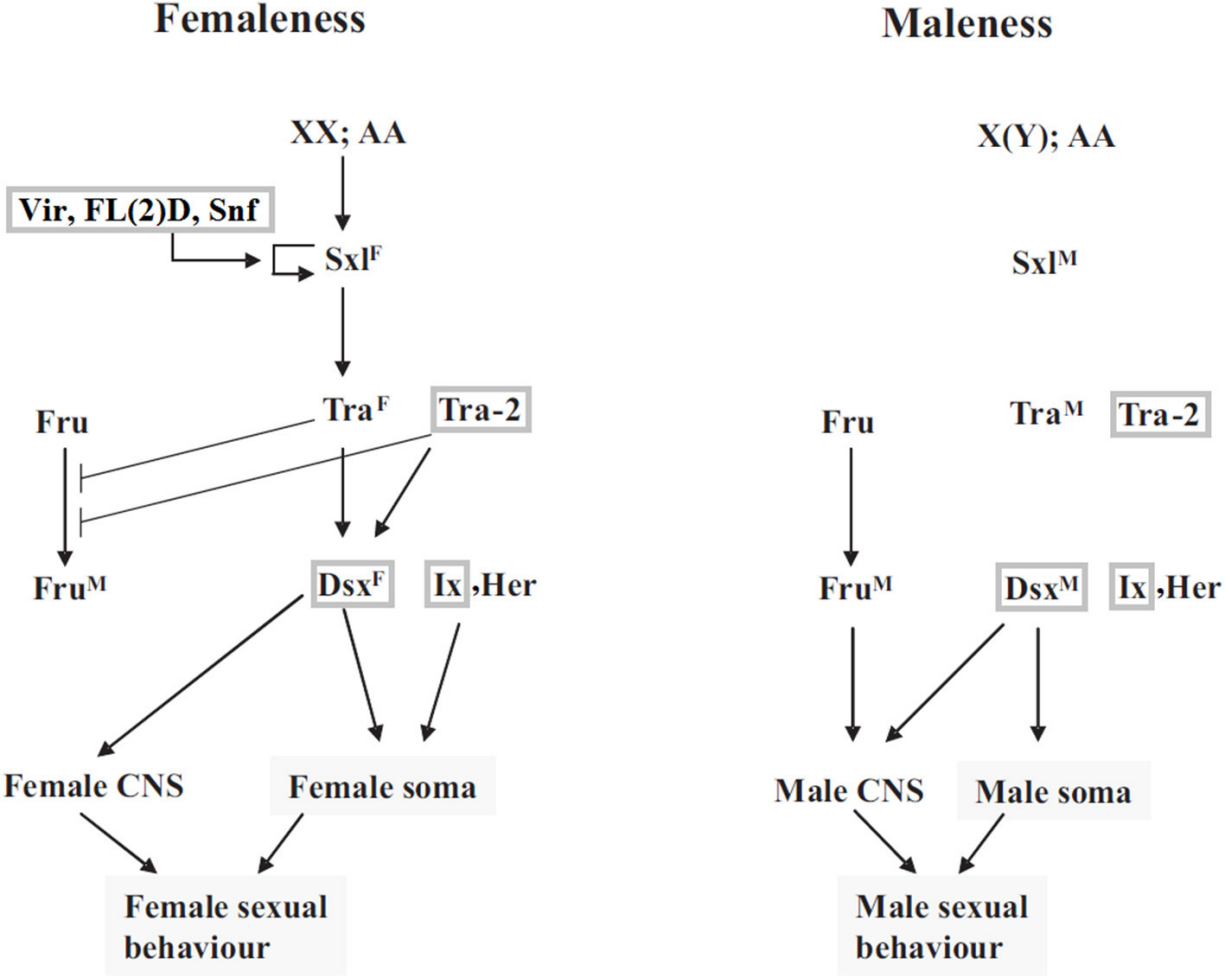
**Sex determination pathway in *Drosophila*, modified from Sánchez (2008)**. Sxl^F^ and Sxl^M^ refer to functional female and nonfunctional male isoforms of the Sxl protein, respectively. Tra^F^ is the functional female form of the Transformer protein, which in conjunction with the constitutive gene product Tra-2 controls female-specific splicing of *dsx* and *fru*. *snf*, *vir* and *FL(2)D* are required for late female-specific splicing of Sxl but play no part in determining early Sxl splicing pattern. The genes for which *Z. pullula* homologs have been identified in this study, are boxed in gray. For more details on the regulation and function of these genes, refer to (Sánchez, 2008; Gempe and Beye, 2011) or other similar resources. Reproduced with permission from The International Journal of Developmental Biology (Int. J. Dev. Biol.) (2008) Vol:52, pp. 837–856.

A number of genes potentially involved in the leafhopper sex determination were identified through homology search with the *Drosophila* sex determination genes. Although pea aphid is *Zyginidia*'s closest relative with a reference genome sequence (The International Aphid Genomics Consortium, 2010), the functional annotation for this genome is not as complete as that of *Drosophila*. Sex determining genes of pea aphid have been found based on homology with *Drosophila* sequences and lack direct experimental verification (The International Aphid Genomics Consortium, 2010). Therefore, we decided to use *Drosophila* sequences as the reference set. Figure 1 depicts the canonical sex determination pathway in *Drosophila*. Homologs of several *Drosophila* sex determination genes were identified among the transcripts including *dsx (doublesex)*, *tra-2 (transformer-2)*, *vir (virilizer)*, *fl(2)d (female lethal d)*, *snf (sans fille)* and *ix (intersex)*. No leafhopper homologs could be identified for *tra (transformer)*, *sxl (sex lethal)*, *fru (fruitless)* or *her (hermaphrodite).* Table 2 shows the expression levels for the identified leafhopper sex determination genes.

**Table 2:** **Homologs of *Drosophila* sex determination genes in the *Zyginidia* transcriptome and their normalized expression levels**.

**Locus**	**Fly homolog**	**Multiple isoforms?**	**FW**	**MW**	**F**	**M**
5652	*dsx*	Y	11.6	49.4	11.7	19.7
8743	*tra-2*	Y	5.9	13.2	8.8	26.1
5015	*vir*	N	20.2	9.9	16.1	23.2
10229	*fl(2)d*	N	17.4	32.9	40.9	63.3
21060	*snf*	Y	0	3.3	20.5	10.9
18743	*ix*	N	5.9	11.6	81.8	43.9

FW, infected female; MW, infected feminized male (intersex female); F, uninfected female; M, uninfected male.

Seventeen genes in our dataset were flagged as likely *Wolbachia* sequences according to the Blast results against the NCBI dataset. Bacterial origin seems very probable for a number of these transcripts based on the expression levels in infected and uninfected lines, plus high similarity to known *Wolbachia* sequences (Table 3). These sequences were Blasted against the aphid genome to check if there was an indication of horizontal transfer; they were also Blasted against the *Drosophila* genome as a distant outgroup (Table 3).

**Table 3:** **Expression levels and aphid and fly homologs of loci whose best Genbank hit was a *Wolbachia* sequence**.

	**Expression**				**Genbank**					**Aphid (*Acyrthosiphon pisum*) homolog**				**Fly (*Drosophila melanogaster*) homolog**			
**Locus**	**FW**	**MW**	**F**	**M**	**Best hit**	**Accession**	**Q %**	**Id %**	**E**	**Accession**	**Q %**	**Id %**	**E**	**Accession**	**Q %**	**Id %**	**E**
1053	146.6	138.2	0	0.2	GroEL [*Wolbachia* endosymbiont of *Bemisia tabaci*]	AFQ62607.1	62	93	5E-142	ACYPI009253	57	37	2E-35	CAA67720.1	57	39	1E-37
1097	51.8	37.9	1.5	0.6	Outer surface protein precursor [*Wolbachia* endosymbiont of *Nasonia vitripennis*]	ABF61215.1	88	83	8E-47	-	-	-	-	-	-	-	-
1331	60.4	36.2	0	0	Molecular chaperone GroeL [*Wolbachia* endosymbiont of *Nasonia vitripennis*]	WP 010401204.1	99	99	9E-75	ACYPI009253	99	44	7E-24	CAA67720.1	97	48	4E-26
13961	34.6	29.6	1.5	1.0	30S ribosomal protein S12 [*Wolbachia* endosymbiont of *Culex quinquefasciatus*]	WP 007301994.1	93	100	2E-59	ACYPI007219	83	53	2E-24	NP_525050.1	75	52	4E-20
22635	0	8.3	40.9	55.1	Ankyrin motif protein [*Wolbachia* endosymbiont of *Cadra cautella*]	BAH22252.1	62	49	6E-92	ACYPI21453	31	33	1E-09	NP_648366.2	24	31	3E-09
23163	0	3.3	0	17.4	Ankyrin domain protein [*Wolbachia pipientis*]	AEX55220.1	93	36	2E-26	ACYPI38268	88	33	9E-21	NP_787123.1	87	31	8E-23
2326	11.6	14.8	1.5	4.1	SD27140p [*Wolbachia* endosymbiont of *Drosophila ananassae*]^*^	WP 007550719.1	53	58	2E-26	ACYPI56754	98	43	1E-29	CAA09069.1	93	40	1E-22
2673	86.3	39.5	0	0.3	Hypothetical protein [*Wolbachia pipientis*]	WP 006013682.1	98	68	4E-55	-	-	-	-	-	-	-	-
26930	0	9.9	22.0	5.0	Ankyrin repeat domain protein [*Wolbachia* endosymbiont of *Nasonia vitripennis*]	WP 010405254.1	61	44	1E-13	ACYPI38268	65	44	9E-14	NP_001246787.1	60	42	3E-14
276	175.4	187.6	17.6	7.6	Unnamed protein product [*Wolbachia* endosymbiont of *Callosobruchus chinensis*]	BAC22720.1	65	41	4E-49	ACYPI28967	19	40	4E-12	CAC16870.1	64	29	3E-31
2876	0	6.6	7.3	7.9	MULTISPECIES: ankyrin [*Wolbachia*]^**^	WP 007302786.1	99	35	2E-19	ACYPI000387	99	35	1E-19	AHN57996.1	99	33	5E-19
295	0	19.8	0	0	Hypothetical protein [*Wolbachia pipientis*]^***^	WP 019236989.1	46	99	6E-103	-	-	-	-	-	-	-	-
3433	69.1	23.1	0	0	Hypothetical protein [*Wolbachia* endosymbiont of *Cadra cautella*]	BAH22204.1	66	84	1E-24	-	-	-	-	-	-	-	-
345	71.9	13.2	3.0	0	Ankyrin motif protein [*Wolbachia* endosymbiont of *Cadra cautella*]	BAH22317.1	100	47	5E-17	ACYPI001311	95	35	1E-08	NP_608900.3	78	34	9E-08
4382	51.8	39.5	0	0	Cold-shock domain family protein [*Wolbachia* endosymbiont of *Drosophila simulans*]	WP 015588193.1	50	99	4E-40	ACYPI000791	40	45	2E-11	AAL28370.1	40	47	1E-10
5403	51.8	32.9	7.3	1.2	Hypothetical protein [*Wolbachia* endosymbiont of *Drosophila simulans*]	WP 015588107.1	99	97	1E-62	-	-	-	-	-	-	-	-
5490	26.0	46.1	0	0	Heme biosynthesis protein HemY [*Wolbachia pipientis*]	WP 006014258.1	99	96	5E-60	-	-	-	-	NP_651267.1	41	35	1E-07

Aphid and fly homologs were identified by Blasting the sequence on Genbank using the species inclusion option. Aphid hits were double-checked by Blasting on Aphidbase.com. Accession number for aphid homologs are from Aphidbase. Other accession numbers are from Genbank. A hit is reported only if the Expected value is <1E-5. Q%, Query coverage percent; Id%, Identity percent; E, Expected value.

* The second best hit is the reported Wolbachia sequence. The first hit is >ref|XP_005192252.1|PREDICTED: uncharacterized protein LOC101899042, partial [Musca domestica].

** The second best hit is the reported Wolbachia sequence. The first hit is >ref|WP_012472427.1|hypothetical protein [Candidatus Amoebophilus asiaticus].

*** Although the best hit the reported hypothetical protein, the second best hit and many after that are annotated as (putative) transposases.

### Principal component analysis of transcriptomes

**Figure 2:**
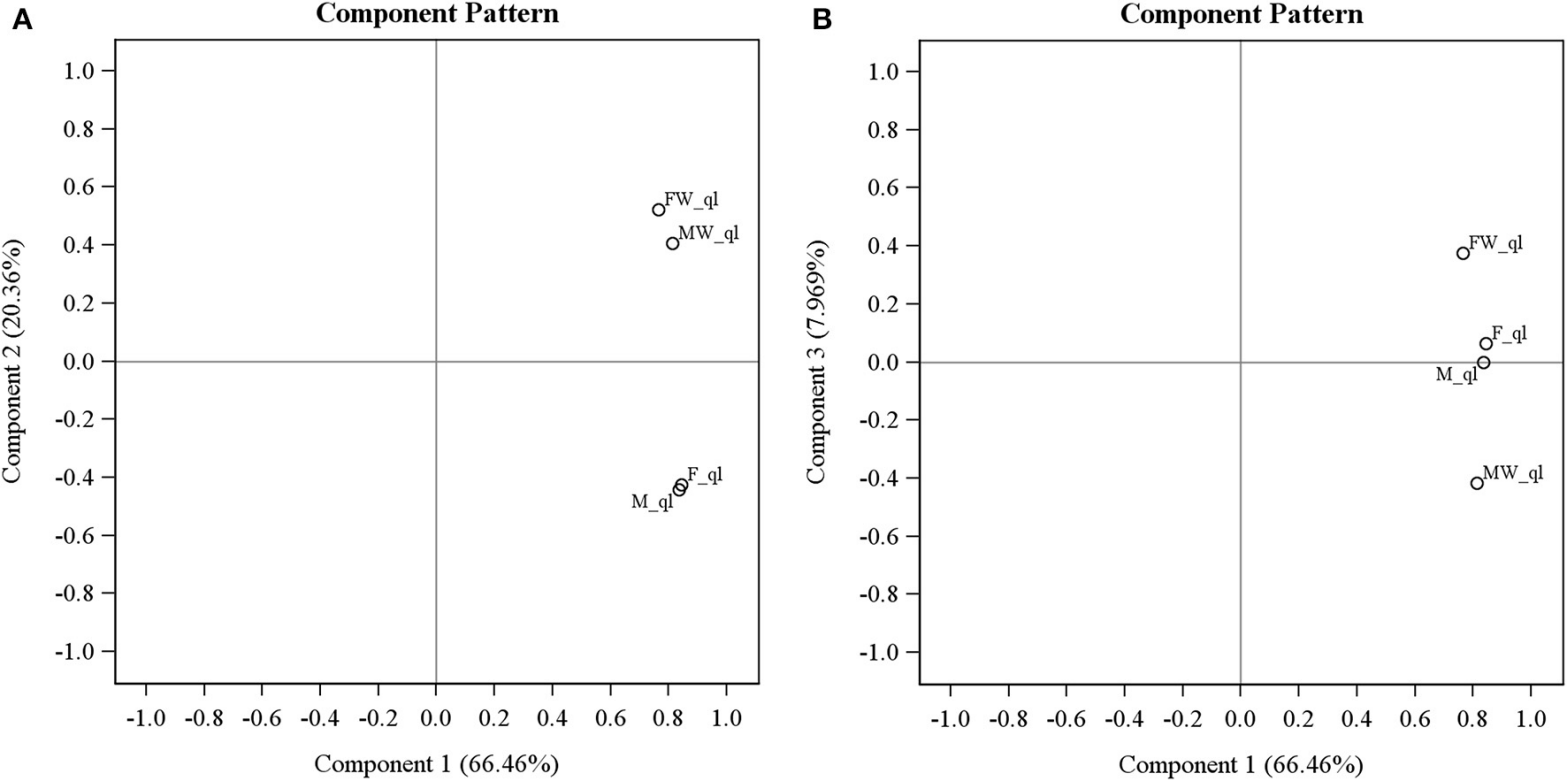
**Principal component analysis of gene expression levels in four *Zyginidia* samples: infected females (FW), infected males or intersex females (MW), uninfected females (F) and uninfected males (M)**. The first three PCs are depicted here. PC1 is correlated with all sample labels but PC2 separates the infected and uninfected samples conclusively. PC3 reveals an interaction term between *Wolbachia* status and sexual karyotype. The “_ql” suffix after line names means that the expression values were quantile-normalized and log transformed. **(A)** PC1 vs. PC2; **(B)** PC1 vs. PC3.

Principal component analysis on transcriptomes of the four leafhopper samples surprisingly revealed that *Wolbachia* infection changes the host transcriptome extensively and the effect is by no means limited to sex-reversal. As evident in Figure 2, the first PC (explaining 66.46% of variance) is highly correlated with all of the samples indicating that the expression of most genes is not significantly altered by *Wolbachia* and is similar across all samples. By the second PC (explaining 20.36% of variance), *Wolbachia* infected male and female samples cluster together and uninfected male and female cluster together. This PC is generated by genes whose expression is changed by *Wolbachia* consistently regardless of sexual karyotype or phenotype. The third PC (explaining 7.97% of variance) indicates an interaction term: F and M are similar and stand in the middle of the scale, with MW and FW occupying the opposite sides of them. This PC is generated by genes that are expressed similarly in uninfected males and females, and *Wolbachia* infection changes their expression in opposite ways in chromosomal males and females. Overall, sex inversion does not seem to be the only or even the biggest effect of *Wolbachia* on gene expression patterns in *Zyginidia*, even if it is the most conspicuous phenotypic consequence; otherwise, we would expect the three phenotypically female groups (F, FW and MW) to cluster together and the only male group (M) to stand separate from them. None of the PCs show such a pattern. PCA was repeated on expression values without the initial outlier filtering, and applying several different normalization and transformation strategies; they all yielded the same picture as described above: the main effect was invariably the presence or absence of *Wolbachia* regardless of sex (details not shown).

## Discussion

We assembled the *Z. pullula* transcriptome *de novo* and produced 18,147 loci and 27,236 transcripts with a total consensus sequence length of 16.3 Mb. These numbers were well within the expected range based on the aphid genome information. The aphid genome was reported to contain 11,089 highly supported RefSeq gene models with a total exonic length of 21.6 Mb; adding the gene models from six other gene prediction programs, a total of 34,604 non-redundant gene models with the total exonic length of 35.7 Mb were described (The International Aphid Genomics Consortium, 2010). The true number of genes is purportedly a number between those two estimates. Hence, our *de novo* assembly of the transcriptome seems to have captured a reasonable proportion of the expressed genes.

The results of sequence homology search (Table 1) confirm the closer relatedness of *Z. pullula* to *A. pisum* (the aphid) than to *Drosophila*. A caveat to this analysis is the extensive set of duplications in the aphid genome (The International Aphid Genomics Consortium, 2010). Without a leafhopper reference genome, we do not know if the same wave of duplications has affected *Z. pullula* or not; however, there was an indication in our data that it might have. By visual inspection of the sequences that were annotated as isoforms of a single locus computationally, we realized that some of them did not show signatures of known alternative splicing patterns; but looked like highly differentiated alleles (details not shown). These may indeed be paralogous sequences in the process of divergence. Further investigation, including the sequencing of single individuals rather than pools of them, will be required to separate paralogy from allelic variation.

A number of leafhopper sex determination genes were identified based on homology with fly sequences (Table 2). Insect sex determination machinery has evolved around the *transformer-doublesex* axis (Sánchez, 2008); *tra* is the fast evolving component responsible for receiving the signal–sometimes through mediators- from the upstream sex determining factors (chromosomal constituent, incubation temperature, etc.), and *dsx* is the conserved switch that relays this signal down to the developmental processes (Sánchez, 2008; Verhulst et al., 2010). It is, therefore, not surprising that we found a homolog for *dsx* and not for *tra* in our dataset. The short length of the aligned segments prevented reliable assignment of male and female isoforms; but these initial results can be used to design primers to extract the whole genes from the leafhopper genome. Future experiments can then follow the flow of the signal in the sex determination pathway to identify where the cascade is diverted to female development in *Wolbachia*-infected genetic males. In the moth *O. scapulalis*, the impact point is somewhere above the level of *dsx* (Sugimoto and Ishikawa, 2012). Having the sequences of *dsx* male and female isoforms, one could check whether this is also true in leafhoppers. Unfortunately, the lack of replicates in our preliminary data makes it impossible to assess the significance of differential expression of genes across our four groups (FW, MW, F, and M). This is another task that remains to be done in future projects. In addition, development of X-linked sequence markers will enable early sexing of the embryos (based on the female XX / male XO karyotypes) through quantitative PCR; and facilitate the study of early developmental processes in infected and uninfected specimens.

We found a number of *Wolbachia*-related transcripts in the sequenced cDNA libraries (Table 3). The loci expressed mainly in infected lines with great similarity to known *Wolbachia* sequences are likely to have *Wolbachia* origin (e.g., loci 1053, 1097, 1331, and 13961). Curiously, a couple of loci are expressed primarily in the uninfected lines (e.g., locus 22635). At this point, we do not have a hypothesis as to the reason behind this observation. Repeating the experiments with replicates and higher sequencing depth would be the first step to confirm the reproducibility of these patterns. Our protocol of mRNA purification for creation of cDNA libraries involved a hybridization step with oligo-T ligands, which targets the eukaryotic mRNA poly-A tails; therefore, it will be necessary to employ a different purification strategy in order to capture most of the poly-A lacking bacterial mRNAs. Table 3 shows that several of the *Wolbachia*-related sequences code for Ankyrin-repeat proteins. *Wolbachia* genomes are well known for containing an extraordinarily high number of these genes (Wu et al., 2004; Iturbe-Ormaetxe et al., 2005). Gene transfer between *Wolbachia* and mosquito hosts has been previously reported (Woolfit et al., 2009). PCR experiments and phylogenetic analyses have confirmed horizontal gene transfer from bacterial endosymbionts to the aphid genome (The International Aphid Genomics Consortium, 2010). Similar approaches will be required to confirm bacterial or insect origin for the transcripts listed in Table 3. We tried to check for possible aphid lineage-specific horizontal transfers by asking whether a likely *Wolbachia* transcript shows high sequence similarity to an aphid sequence, but not a fly sequence; none of the loci in Table 3 expressed such a pattern. One of the *Wolbachia*-related transcripts showed a degree of homology with the aphid *vasa* gene (locus 4382). Almost identical homologs of this sequence exist in the three published *Wolbachia* genomes (Blast results not shown); its homologs in fly, leafhopper and the published *Wolbachia* genomes are characterized or predicted ATP-dependent RNA helicases. *vasa* has been implicated in transmission of maternal effects and sex determination in clams (Milani et al., 2011). It will be very interesting to check if products of host-homologous genes are actually exported out by *Wolbachia* into the host cell.

We used natural isolates of infected and uninfected leafhoppers for our comparisons with no antibiotic treatment. This relieved our comparisons from the confounding effects of antibiotic treatments on the host physiology. The rationale behind the traditional use of antibiotics to cure the infected lines from *Wolbachia* is to obtain infected and uninfected lines with the same genetic background. However, antibiotics can change the host physiology substantially, and quite remarkably, their effect can perpetuate through several generations of unexposed progeny (Ballard and Melvin, 2007; Zeh et al., 2012; Fridmann-Sirkis et al., 2014). We avoided the use of antibiotics completely and achieved homogenous genetic backgrounds among samples by taking advantage of repeated backcrossing of infected females to uninfected males. We collected all of our founder specimens from the same leafhopper population in a grass field. In the sampled population, the sex-ratio was only moderately female biased, with a moderate prevalence of the infection (~1:1.8 male:female, *Wolbachia* infection rate ~30% of the collected females; Negri I., unpublished data). As uninfected males are the only physiological males in existence, all the “egg-laying females” (in the field and in the lab, including the females used in this study) always mate with (and only with) uninfected males. Thus, all of our infected and uninfected lines come from the same genetic background. We carried out three further generations of backcrossing of infected females to uninfected males in the lab to effectively remove any residual genetic variation between the two groups. Details of rearing conditions are described in Negri et al. (2006). The natural pattern of sexual reproduction and the additional backcrossing done in the lab ensure the similarity of nuclear genetic backgrounds. We also tested mitochondrial gene sequences in *Zyginidia* samples from different Italian localities, both infected and uninfected, and they were all nearly identical (Negri I., unpublished data).

Through principal component analysis, we have showed that *Wolbachia*-induced changes in the host transcriptome are mainly sex-independent, and cannot be explained only by the sex reversal of genetic males. Previous transcriptomic studies on *Wolbachia* have reported changes in the expression of genes unrelated to the reproductive phenotype. For instance, *Wolbachia* infection in *Armadillidium vulgare* triggered the overexpression of immune-related genes (Chevalier et al., 2012). In the parasitoid wasp *Asobara tabida*, endosymbiont infection or lack thereof was associated with changes in expression of genes related to female reproductive development, iron and oxidative stress regulation, and immune recognition (Kremer et al., 2009, 2012). Artificial infection of *Anopheles* cell cultures by *Wolbachia*, surprisingly caused down-regulation of immune, stress response and detoxification genes (Hughes et al., 2011). *Wolbachia*-inoculated *Drosophila* cell lines exhibited differential expression of several GO categories not directly related to reproduction, including antimicrobial humoral response, ion homeostasis, response to unfolded protein and response to chemical stimulus (Xi et al., 2008). In *Aedes aegypti*, *Wolbachia* was shown to manipulate the expression of a metalloprotease gene through induction of a specific host miRNA (Hussain et al., 2011). Apart from such direct evidence, the observation of various forms of fitness cost in the feminized males, is consistent with the idea that sex reversal is not the sole effect of feminizing *Wolbachia* (Moreau et al., 2001; Rigaud and Moreau, 2004). Nevertheless, our study is the first one to quantitatively demonstrate that infection itself has a larger effect than that of sex reversion, through PCA of all of the available gene expression levels.

Lack of replicates meant that we could not quantitatively identify differentially expressed genes between the lines because we could not calculate variances. Instead, we focused on the global patterns of gene expression by applying PCA to gene expression values. Thousands of loci (each acting as one observation point) were used to generate the PCs. Antibiotic treatment and different genetic backgrounds could have been two potential sources of systematic bias in this type of analysis; they could have generated similar clustering patterns and confounded the interpretation of results. However, through the single-population sampling and the repeated backcrossing scheme, we avoided both sources of confusion.

Based on the PCA results, we encourage the use of biochemical bottom-up approaches focusing on the whole *Wolbachia* effect rather than the specific sex inversion event. *Wolbachia*'s effect is perceivably mediated by molecules secreted into the host cell or expressed on the outer membrane surface of the bacterium-containing vesicles. *Wolbachia* cannot be maintained in cell-free cultures indefinitely; but there are protocols to keep them alive in synthetic media for several hours (Rasgon et al., 2006; Gamston and Rasgon, 2007). In such a setting, the molecules released into the medium can be detected and purified using chromatographic and/or mass spectrometric approaches. Appropriate methods can be used, too, for isolation and characterization of surface molecules from the bacterium-containing vesicles. Pull-down experiments on the host proteins by these *Wolbachia* released or surface molecules might reveal the initial cellular targets of the endosymbiont-host interaction.

### Conflict of interest statement

The authors declare that the research was conducted in the absence of any commercial or financial relationships that could be construed as a potential conflict of interest.
